# Initial validation of a self-report questionnaire based on the Theoretical Domains Framework: determinants of clinician adoption of a novel colorectal cancer screening strategy

**DOI:** 10.1186/s43058-021-00221-x

**Published:** 2021-10-19

**Authors:** Xuan Zhu, Minji K. Lee, Emily Weiser, Joan M. Griffin, Paul J. Limburg, Lila J. Finney Rutten

**Affiliations:** 1 Robert D. and Patricia E. Kern Center for the Science of Healthcare Delivery, Mayo Clinic, 200 First Street SW, Rochester, MN USA; 2 Exact Sciences Corporation, Madison, WI USA; 3 Division of Health Care Delivery Research, Mayo Clinic, Rochester, MN USA; 4 Division of Gastroenterology and Hepatology, Mayo Clinic, Rochester, MN USA; 5 Division of Epidemiology, Mayo Clinic, Rochester, MN USA

**Keywords:** Colorectal cancer screening, Theoretical domains framework, Implementation, Questionnaire validation

## Abstract

**Background:**

Colorectal cancer (CRC) screening for average risk adults age 45 and older continues to be underutilized in the USA. One factor consistently associated with CRC screening completion is clinician recommendation. Understanding the barriers and facilitators of clinical adoption of emerging CRC screening strategies is important in developing effective intervention strategies to improve CRC screening rates. We aimed to develop a questionnaire based on the Theoretical Domains Framework (TDF) to assess determinants of clinical adoption of novel CRC screening strategies, using the multi-target stool DNA test (mt-sDNA; Cologuard®) as an example, and test the psychometric properties of this questionnaire on a sample of US clinicians.

**Methods:**

A web survey was administered between November and December 2019 to a national panel of clinicians including primary care clinicians (PCCs) and gastroenterologists (GIs) to assess 10 TDF constructs with 55 items. Confirmatory factor analysis (CFA) was used to examine whether the a priori domain structure was supported by the data. Discriminant validity of domains was tested with Heterotrait-Monotrait ratio (HTMT). Internal consistency for each scale was assessed using Cronbach’s alpha. Criterion validity was assessed with self-reported mt-sDNA use and mt-sDNA recommendation as the outcomes.

**Results:**

Complete surveys were received from 814 PCCs and 159 GIs (completion rate, 24.7% of 3299 PCCs and 29.6% of 538 GIs). Providers were excluded from analysis if they indicated not recommending CRC screening to average-risk patients (final *N* = 973). The final questionnaire consisted of 38 items covering 5 domains: (1) knowledge; (2) skills; (3) identity and social influence; (4) optimism, beliefs about consequences, and intentions; and (5) environmental context and resources. CFA results confirmed a reasonable fit (CFI = 0.948, SRMR = 0.057, RMSEA = 0.080). The domains showed sufficient discriminant validity (HTMT < 0.85), good internal consistency (McDonald’s omega > 0.76), and successfully differentiated providers who reported they had ordered mt-sDNA from those who never ordered mt-sDNA and differentiated providers who reported routinely recommending mt-sDNA from those who reported not recommending mt-sDNA.

**Conclusions:**

Findings provide initial evidence for the validity and internal consistency of this TDF-based questionnaire in measuring potential determinants of mt-sDNA adoption for average-risk CRC screening. Further investigation of validity and reliability is needed when adapting this questionnaire to other novel CRC screening strategy contexts.

**Supplementary Information:**

The online version contains supplementary material available at 10.1186/s43058-021-00221-x.

Contributions to the literature • We developed and evaluated a questionnaire based on the Theoretical Domains Framework to measure potential determinants of clinician adoption of novel colorectal cancer (CRC) screening strategies, using the multi-target stool DNA test (mt-sDNA; Cologuard®) as an example.

• This questionnaire provides researchers and implementers a way to reliably assess theoretically grounded factors that shape clinician beliefs and behaviors surrounding average-risk CRC screening strategies.

• Findings from this questionnaire can aid in identifying determinants of successful implementation of novel CRC screening strategies in clinical practices and inform the development and evaluation of clinician interventions to improve CRC screening rates among average risk patients.

## Background

Colorectal cancer (CRC) is the second leading cause of cancer-related deaths in the USA among women and men combined [1, 2]. Major guideline organizations recommend CRC screening among average-risk adults age 45–75 or 50–75 and recommend multiple stool-based and visualization-based screening options [3–5]. However, CRC screening continues to be underutilized in the USA, with nearly one third of eligible adults reportedly not up-to-date [6]. One factor consistently associated with higher CRC screening rates is clinician recommendation [7–9]. Given the availability of multiple screening strategies that differ in attributes such as safety, efficacy, cost, and the availability of patient navigation, clinicians are increasingly encouraged to engage patients in shared decision-making to select a strategy that is consonant with patient needs and preferences [3, 10–14]. As new CRC screening strategies emerge, understanding how various factors shape clinicians’ adoption of screening strategies is important in developing effective intervention strategies to improve CRC screening rates.

Behavior change theories are particularly useful for guiding the examination of factors shaping clinician behaviors regarding CRC screening because they offer hypotheses about the conditions under which (*when*) and the mechanisms through which (*why*) behavior change happens. Understanding *when* and *why* behavior change happens can inform the design of implementation strategies to optimize conditions for behavior change and inform the evaluation of implementation processes and outcomes. Theoretical domains framework (TDF) [15] is an integrated theoretical framework that synthesized 33 behavior theories into 14 construct domains that influence behavior change in the implementation context [15–17]. TDF has been used to identify determinants of behavior change, select behavior change techniques, design intervention strategies, and evaluate the implementation process across a wide range of clinical contexts and behaviors [15–24].

To our knowledge, a questionnaire examining determinants of clinician adoption of emerging CRC screening strategies has not been documented in the literature. Having a comprehensive, valid instrument to reliably assess theoretically grounded factors that shape clinician behaviors surrounding CRC screening could aid in identifying determinants of successful implementation of novel CRC screening strategies and inform organizational and provider-level interventions to improve CRC screening rates. Therefore, we aimed (1) to develop a TDF-based questionnaire to assess determinants of clinician adoption of novel CRC screening strategies using the multi-target stool DNA test (mt-sDNA; Cologuard®, a stool-based CRC screening option for average-risk adults age 45 and older) as an example, and (2) to test the psychometric properties of this questionnaire on a sample of US clinicians. We address four specific research questions: (1) Do the data support the pre-defined TDF-based structure of the items (i.e., construct validity)? (2) To what extent are the domains distinct from each other (i.e., discriminant validity)? (3) How well do items intended to measure the same domain actually measure the same domain (i.e., internal consistency)? And (4) how well do the domains differentiate clinicians who have used (or routinely recommended) mt-sDNA and those who have not used (or routinely recommended) mt-sDNA (i.e., criterion validity)?

## Methods

### Survey development

We developed a 55-item questionnaire based on Huijg et al. [25, 26] to assess the following 10 TDF domains: knowledge; skills; professional role and identity; social influences; beliefs about capabilities; intentions; optimism; beliefs about consequences; memory, attention, and decision processes; and environmental context and resources. We excluded 3 domains (goals, reinforcement, behavioral regulation) based on published research showing that these items lacked discriminant validity [25, 26]. We also excluded the emotion domain because it is less relevant to our context. We modified Huijg et al. [25, 26] items and included additional items to fit the Cologuard use context. Additional file 1: Appendix summarizes the items and their TDF domain assignment. All items were measured on a 5-point Likert scale (1 = strongly disagree to 5 = strongly agree). We referred to mt-sDNA as Cologuard®, as it is the only mt-sDNA test currently approved by the FDA for clinical application. Additional measures including whether the provider had ever ordered mt-sDNA for a patient (mt-sDNA use) and whether the provider routinely recommended mt-sDNA for CRC screening to average-risk patients (mt-sDNA recommendation) [27–29]. Pretest interviews were conducted with 11 clinicians from the survey panel to validate the survey length, survey programming, and data collection methodology prior to administering the survey.

### Data collection

**Fig. 1:**
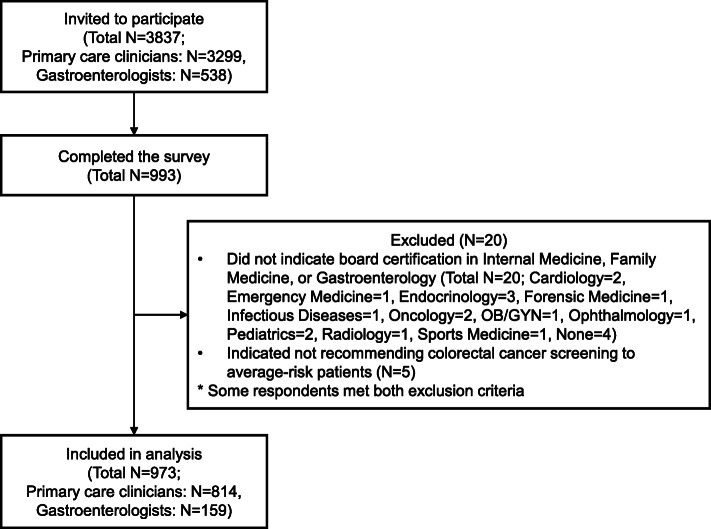
Study flowchart

Data were collected through a web survey between November and December 2019 by AmeriSpeak®, a research panel developed and funded by NORC at the University of Chicago (http://www.norc.org) using a third-party vendor, Dynata, who maintains a validated panel of over 200,000 US clinicians. Among 3299 primary care clinicians (PCCs) and 538 gastroenterologists (GIs) who were invited, 993 clinicians (26%) completed the survey. Providers who indicated specializations other than internal medicine, family medicine, or gastroenterology (e.g., pediatrics, cardiology; *N* = 20) or indicated not recommending CRC screening to average-risk patients (*N* = 5) were excluded, resulting in a final sample of 814 PCCs (24.7%) and 159 GIs (29.6%) (Fig. 1). Participants received remunerations based on fair market value hourly rate ($39 for PCCs and $51 for GIs).

### Analysis

To examine whether the a priori assignment of the items based on TDF is supported by the data, we used confirmatory factor analysis (CFA) with robust weighted least squares estimation (WLSMV), a method suitable for ordinal data [30–32]. Model fit was assessed using multiple criteria based on recommendations in the literature [33–35].

To reduce the risk of capitalization on chance, we split the full sample randomly into two subsamples of equal size [36]. We used subsample 1 to generate and refine the model and we used subsample 2 to validate the resulting model. Because the fit of the initial 10-domain model was poor, we switched to an exploratory approach. We first examined the polychoric correlation matrix of the items for values exceeding .85 which would suggest one of the two corresponding items is redundant. We then conducted parallel analysis to identify the number of major factors in the data and used hierarchical agglomerative cluster analysis to examine item clusters [37–39]. Redundant items and items that did not map onto the intended domains were removed. We re-specified a CFA model based on the exploratory analyses and tested it with subsample 1. To improve model fit, we examined the residual covariance matrix to identify areas that are not well-explained by the model, then correlated the error terms of items that are conceptually related or similar in question wording to account for the method effect. This step was done iteratively; we examined model fit after each adjustment and stopped adjustment once acceptable fit was achieved. The final model was then tested with subsample 2.

Discriminant validity of the domains was assessed using heterotrait-monotrait ratio of correlations (HTMT), with a value < 0.85 considered satisfactory [40, 41]. The internal consistency of each domain scale was assessed using McDonald’s omega [42].

After a theoretically meaningful and statistically acceptable factor model was achieved, we derived domain scores for each provider by generating factor scores (i.e., estimated values for the latent variables) from the final CFA model and rescaling them to have a 1–5 range to facilitate interpretation. To assess criterion validity, we examined whether the domains can predict mt-sDNA use (i.e., whether the provider had ever ordered mt-sDNA) and mt-sDNA recommendation (i.e., whether the provider routinely recommended mt-sDNA for CRC screening) using logistic regression. Additionally, we conducted two multivariate analysis of variance (MANOVA) with the domain scores as the outcomes and mt-sDNA use and mt-sDNA recommendation as the independent variable in each model to understand how providers’ domain scores differ by mt-sDNA use and mt-sDNA recommendation.

## Results

### Sample characteristics

Sample characteristics were reported in Table 1. Among PCCs, 77% had ever ordered mt-sDNA for a patient, compared to 78% of GIs. A minority of PCCs (23%) and GIs (22%) indicated that they do not routinely recommend mt-sDNA for average-risk CRC screening. For cross-validation purposes, we split the full sample randomly into exploratory subsample 1 (*N* = 486) and confirmatory subsample 2 (*N* = 487). The two subsamples did not differ on provider characteristics.

**Table 1:** Provider and practice characteristics of participants by specialty

	Primary care clinicians^a^ (*N* = 814)	Gastroenterologists (*N* = 159)
	*N* (%)	*N* (%)
Age in years		
27–39	107 (13.1)	41 (25.8)
40–49	254 (31.2)	42 (26.4)
50–59	236 (29.0)	45 (28.3)
60 and older	217 (26.7)	31 (19.5)
Sex^b^		
Male	586 (72.2)	131 (82.9)
Female	226 (27.8)	27 (17.1)
Race/ethnicity		
White, non-Hispanic (NH)	534 (65.6)	88 (55.4)
Black, NH	19 (2.3)	4 (2.5)
Hispanic	26 (3.2)	10 (6.3)
Asian/Pacific Islander, NH	193 (23.7)	42 (26.4)
Other/multiple race, NH	42 (5.2)	15 (9.4)
Annual household income		
Less than $74,999	43 (5.3)	4 (2.5)
$75,000 to $124,999	104 (12.8)	9 (5.7)
$125,000 to $174,999	115 (14.1)	12 (7.6)
$175,000 to $199,999	86 (10.6)	16 (10.1)
$200,000 or more	466 (57.2)	118 (74.2)
Board certification		
Internal medicine	387 (47.5)	--
Family medicine	427 (52.5)	--
Gastroenterology	0 (0)	159 (100)
Number of years practicing medicine post-residency		
0–9	116 (14.3)	42 (26.4)
10–19	277 (34.0)	53 (33.3)
20–29	271 (33.3)	45 (28.3)
30+	150 (18.4)	19 (12.0)
Average number of patients seen on typical day		
0–15	163 (20.0)	41 (25.8)
16–20	291 (35.7)	49 (30.8)
21–25	188 (23.1)	30 (18.9)
> 25	172 (21.1)	39 (24.5)
Number of clinicians in practice		
1–15	591 (72.6)	103 (64.8)
16+	223 (27.4)	56 (35.2)
Characterization of clinical practice location		
Urban	262 (32.2)	81 (50.9)
Suburban	447 (54.9)	69 (43.4)
Rural	105 (12.9)	9 (5.7)
Ever ordered mt-sDNA for a patient in their care		
Yes	583 (71.6)	128 (80.5)
No	231 (28.4)	31 (19.5)
Routinely recommend mt-sDNA for CRC screening to average risk patients		
Yes	628 (77.1)	124 (78.0)
No	186 (22.9)	35 (22.0)

^a^Includes internal medicine and family medicine

^b^Missing = 2 for primary care clinicians, missing = 1 for gastroenterologists

### Analysis on exploratory subsample 1

**Fig. 2:**
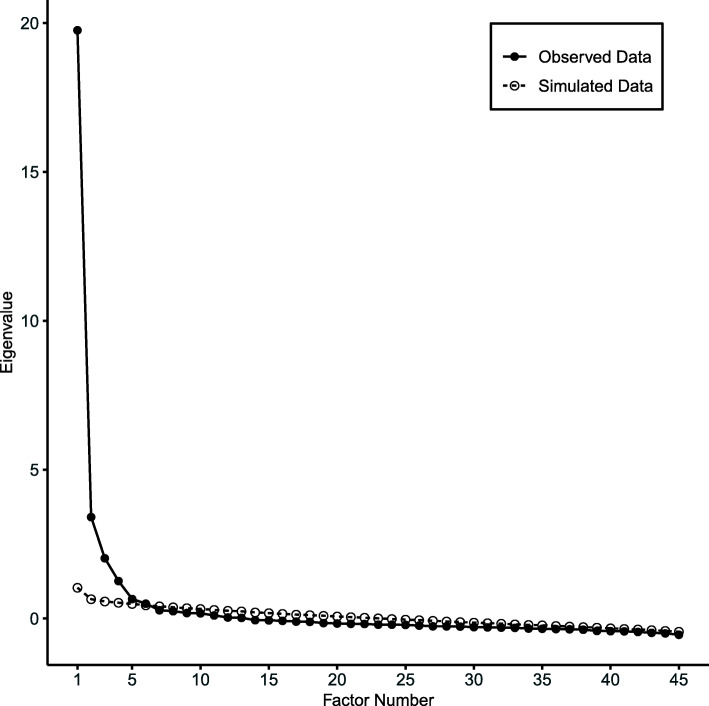
Parallel analysis scree plot. The scree plot shows the eigenvalues (variance explained) of each potential factor extracted from the polychoric correlation matrix of the data. The scree plot suggests that the data may have 5 major factors because the slope started levelling off after the 5th factor (5th black circle). The parallel analysis compared the eigenvalues from the actual data with the eigenvalues from randomly generated data. The parallel analysis suggests that the eigenvalues of the 5 factors based on the actual data are larger than the eigenvalues of these factors that are based on the randomly generated data (the first 5 black circles are above the first 5 white circles)

**Fig. 3:**
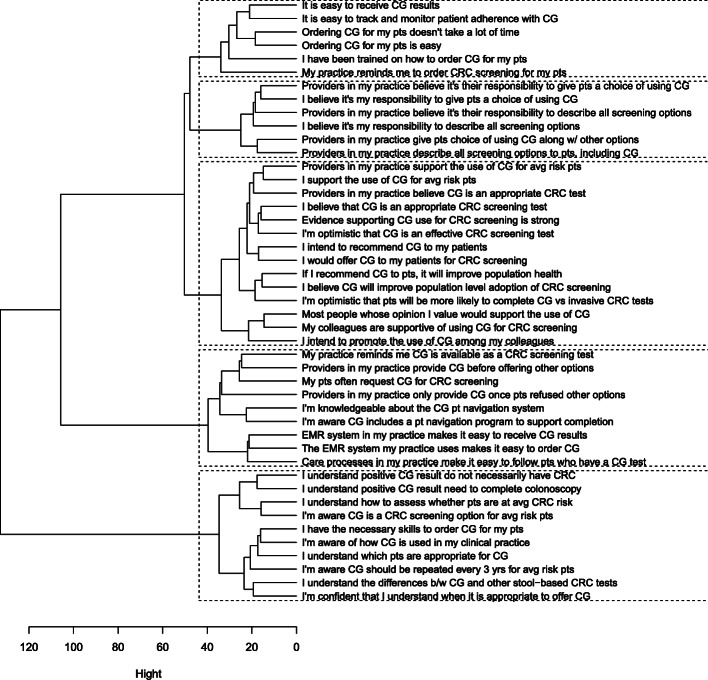
Cluster dendrogram. The dendrogram shows the hierarchical clustering of the items. The height (indicated by the *x*-axis) at which any two items/clusters are joined together indicates the closeness between two items/clusters. The shorter the height, the closer the items/clusters are. The dendrogram was derived from a hierarchical agglomerative cluster analysis performed using Ward’s method with squared Euclidean Distance as the distance measure

CFA results of the initial 10-domain model showed poor fit (Table 2), suggesting significant discrepancies between the observed data and the theorized model. We removed 9 redundant items after examining the correlation matrix and removed 1 item that measures the behavior outcome of mt-sDNA use rather than its antecedent. The parallel analysis and scree plot showed that the data may have 5 major factors (Fig. 2). The cluster analysis (Fig. 3) showed that the 1st factor consists mostly of items about skills and perceived ease of Cologuard use. The 2nd factor consists of items about professional role and social influence, the 3rd factor mostly consists of items about optimism, positive consequences, and intentions regarding mt-sDNA use, representing the reflective and evaluative processes that motivate the performance of a behavior [43]. The 4th factor mostly contains items about environmental context and resources regarding mt-sDNA use and CRC screening. The 5th factor contains mostly items about knowledge regarding Cologuard use and CRC screening. Seven items were removed because they did not cluster with other items of their intended domains. Reasons for item removal were summarized in Additional file 1: Appendix.

**Table 2:** Confirmatory factor analysis fit indexes

Model^a^	*χ*^2^	df	CFI	RMSEA	90% CI of RMSEA		SRMR
					Lower bond	Upper bond	
Initial 10-factor model with subsample 1	8685.30	1385	0.897	0.104	0.102	0.106	0.096
Initial 5-factor model with subsample 1	3077.62	619	0.941	0.090	0.087	0.094	0.064
Revised 5-factor model with subsample 1	2358.83	607	0.958	0.077	0.074	0.080	0.057
Revised 5-factor model with subsample 2	2396.75	607	0.944	0.078	0.075	0.081	0.065
Revised 5-factor model with full sample	4360.58	607	0.948	0.080	0.078	0.082	0.057

^a^Please see appendix 1 for items included in each model

We re-specified a 5-factor CFA model using the remaining 38 items and achieved acceptable fit after allowing the error terms of 12 pairs of items to correlate because they are conceptually related or similar in question wording: *χ*^2^(607) = 2358.83, *p* < .001, CFI = 0.958, SRMR = 0.057, RMSEA = 0.077 (90% CI, 0.074–0.080) (Table 2). All pairs of domains satisfied the criterion for establishing discriminant validity (Table 3) and all domains demonstrated good internal consistency (Table 4).

**Table 3:** HTMT ratios of correlations for each pair of domains (subsample 1/subsample 2/full sample)

	Domain	1	2	3	4	5
1	Knowledge	1				
2	Skills	0.755/0.767/0.761	1			
3	Professional role and social influence	0.561/0.536/0.549	0.692/0.654/0.673	1		
4	Optimism, beliefs about consequences, and intentions	0.579/0.551/0.566	0.755/0.700/0.728	0.746/0.702/0.725	1	
5	Environmental context and resources	0.469/0.441/0.456	0.781/0.781/0.780	0.664/0.622/0.644	0.657/0.577/0.620	1

**Table 4:** Internal consistency of theoretical domains

Domain	Number of items	McDonald’s omega		
		Subsample 1	Subsample 2	Full sample
Knowledge	10	0.911	0.822	0.877
Skills	5	0.860	0.758	0.779
Professional role and social influence	6	0.877	0.866	0.862
Optimism, beliefs about consequences, and intentions	10	0.902	0.890	0.903
Environmental context and resources	7	0.840	0.803	0.819

### Analysis on confirmatory subsample 2

The 5-factor model was confirmed using subsample 2. The fit indexes suggested an acceptable fit between the data and the model, *χ*^2^(607) = 2396.75, *p* < .001, CFI = 0.944, SRMR = 0.065, RMSEA = 0.078 (90% CI, 0.075–0.081) (Table 3). All pairs of domains achieved discriminant validity (Table 4) and all domains demonstrated internal consistency (Table 4).

### Criterion validity

Analysis showed that “Knowledge,” “Optimism, beliefs about consequences, and intentions,” and “Environmental context and resources” were positively associated with mt-sDNA use and mt-sDNA recommendation. Providers with higher scores on these domains were more likely to report having ordered mt-sDNA for a patient (Use: OR = 10.59, 95% CI = 4.49–25.89; OR = 1.89, 95% CI = 1.11–3.23; OR = 2.94, 95% CI = 1.10–7.95; Recommendation: OR = 4.14, 95% CI = 1.86–9.41; OR = 4.05, 95% CI = 2.45–6.76; OR = 2.94, 95% CI = 1.17–7.43) (Table 5). Given the high correlations between the domains (*r* ranges from .535 to .848), the reason the associations between the behavior outcomes and “Skills” and “Professional role and social influence” were not statistically significant could be due to a multicollinearity issue rather than that these domains had little influence on the outcomes.

**Table 5:** Results from logistic regression and differences in mean domain scores by provider mt-sDNA use and mt-sDNA recommendation

Domain	Ever ordered mt-sDNA			Routinely recommend mt-sDNA		
	Logistic regression results	Mean domain scores		Logistic regression results	Mean domain scores	
	OR (95% CI)	No	Yes	OR (95% CI)	No	Yes
		M (SD)	M (SD)		M (SD)	M (SD)
Knowledge	10.59 (4.49–25.89)	2.66 (0.60)	3.63 (0.70)	4.14 (1.86–9.41)	2.78 (0.69)	3.54 (0.75)
Skills	0.44 (0.08–2.39)	2.59 (0.47)	3.41 (0.66)	0.28 (0.05–1.37)	2.64 (0.52)	3.35 (0.68)
Professional role and social influence	0.98 (0.59–1.63)	2.80 (0.58)	3.48 (0.71)	0.91 (0.57–1.46)	2.79 (0.61)	3.45 (0.71)
Optimism, beliefs about consequences, and intentions	1.89 (1.11–3.23)	2.71 (0.56)	3.44 (0.74)	4.05 (2.45–6.76)	2.65 (0.58)	3.42 (0.72)
Environmental context and resources	2.94 (1.10–7.95)	2.68 (0.56)	3.33 (0.69)	2.94 (1.17–7.43)	2.68 (0.58)	3.29 (0.70)

To understand how providers who reported mt-sDNA use differ from those who did not report mt-sDNA use on each theoretical domain, we compared their mean scores of each domain using a 5x2 MANOVA. The result showed a large difference across the five domains (*F*(5, 967) = 83.60, *p* < .001, Pillai’s trace = 0.302, partial *η*^2^ = .302). Providers who reported mt-sDNA use scored statistically significantly higher on each domain compared to providers who reported no mt-sDNA use (“Knowledge”: *F*(1, 971) = 396.13, *p* < .001, *η*^2^ = .290; “Skills”: *F*(1, 971) = 346.15, *p* < .001, *η*^2^ = .263; “Professional role and social influence”: *F*(1, 971) = 195.54, *p* < .001, *η*^2^ = .168; “Optimism, beliefs about consequences, and intentions”: *F*(1, 971) = 212.74, *p* < .001, *η*^2^ = .180) ; “Environmental context and resources”: *F*(1, 971) = 184.94, *p* < .001, *η*^2^ = .160).

**Fig. 4:**
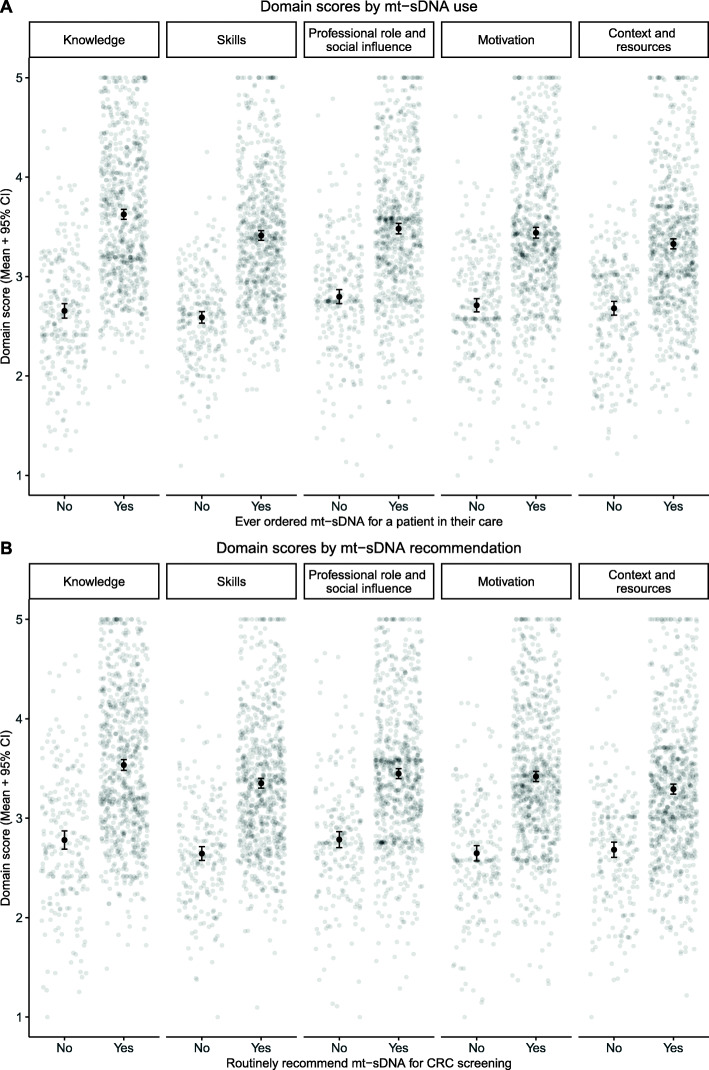
Mean domain scores and 95% CIs by **A** mt-sDNA use and **B** mt-sDNA recommendation

Another 5x2 MANOVA was conducted to understand how providers who reported routinely recommending mt-sDNA versus not recommending mt-sDNA differ on each domain. The results showed a large difference across the five domains (*F*(5, 967) = 49.50, *p* < .001, Pillai’s trace = 0.204, partial *η*^2^ = .204). Providers who reported mt-sDNA use scored statistically significantly higher on each domain compared to providers who reported no mt-sDNA use (“Knowledge”: *F*(1, 971) = 180.12, *p* < .001, *η*^2^ = .156; “Skills”: *F*(1, 971) = 203.03, *p* < .001, *η*^2^ = .173; “Professional role and social influence”: *F*(1, 971) = 158.24, *p* < .001, *η*^2^ = .140; “Optimism, beliefs about consequences, and intentions”: *F*(1, 971) = 212.25, *p* < .001, *η*^2^ = .179); “Environmental context and resources”: *F*(1, 971) = 140.28, *p* < .001, *η*^2^ = .126). Table 5 and Fig. 4 summarizes mean differences in each domain by mt-sDNA use and recommendation.

## Discussion

We developed and tested a TDF-informed questionnaire examining determinants of clinician adoption of novel CRC screening methods using the mt-sDNA test as an example. Our analyses showed that the 10 TDF domains can be described by 5 domains with reasonable to good construct validity, discriminant validity, and internal consistency. The 5 domains include (1) knowledge; (2) skills; (3) identity and social influence; (4) optimism, beliefs about consequences, and intentions; and (5) environmental context and resources. Additionally, the domains demonstrated criterion validity for provider mt-sDNA use and mt-sDNA recommendation. Each subscale successfully differentiated between providers who reported had ordered mt-sDNA for their patients versus providers who had not ordered mt-sDNA and differentiated between providers who reported routinely recommending mt-sDNA for CRC screening versus providers who reported not recommending mt-sDNA. The differences in mean domain scores by mt-sDNA use and recommendation were large [44].

For mt-sDNA use, the “Knowledge” and “Skills” domains showed the largest differences and the “Environmental context and resources” domain showed the smallest difference. Providers who had not ordered mt-sDNA scored, on average, below the scale midpoint (Fig. 4). These findings suggest that there are large rooms for improvement in all 5 domains for our providers who have not used mt-sDNA and intervention strategies targeting the “Knowledge” and “Skills” domains may lead to the largest increase in mt-sDNA use. To develop appropriate intervention strategies to address the target domains, we can start by mapping the domains to the components of the Capability, Opportunity, and Motivation Model of Behaviour (COM-B) and selecting the intervention functions from its accompanying Behavior Change Wheel [43]. In our example, the “Knowledge” and “Skills” domains map onto the “Capacity” component of COM-B and the appropriate intervention functions may include “education,” “training,” and “enablement.” Once potential intervention functions are identified, we can refer to the Behavior Change Technique Taxonomy [45] to select the specific techniques that have been shown to serve the particular intervention functions in previous research and are appropriate for the specific clinical behavior and practice context.

It is worth noting that we developed the questionnaire based on a 10-domain structure while the results showed that a 5-domain structure was more appropriate for our data. The TDF domains “Intentions,” “Optimism,” and “Beliefs about consequences” failed to establish discriminant validity due to high correlations between items across domains. We also combined “Knowledge” and “Beliefs about capabilities” into one domain for the same reason. Previous research has observed similar high correlations across TDF domains in other clinical contexts [21, 46, 47], suggesting the domains are not each describing a unique aspect of a behavior. If the goal is to identify TDF domains that are most influential for behavior change in a given context, high correlations between domains may limit researchers’ ability to take a multivariate approach to examine the associations between the domains and the behavior outcome.

To our knowledge, this is the first questionnaire aimed to assess theoretically grounded determinants of clinician adoption of novel CRC screening strategies. Data collected through this questionnaire can be used to ascertain barriers and facilitators to clinician adoption of novel CRC screening tests, such as mt-sDNA, and to help identify appropriate implementation strategies [48]. It should be noted that a TDF-informed questionnaire can aid in identifying determinants of a specific implementation behavior in a given context, but it does not provide directions on *how* to bring about behavior change as TDF does not specify the causal relationships between domains. Therefore, researchers need to consult additional behavior change theories [43, 49, 50] relevant to the selected domains to identify the mechanisms through which behavior change occur and choose intervention strategies accordingly.

### Limitations

First, due to the small sample size of GIs, we were unable to examine whether the questionnaire’s psychometric properties differ between PCCs and GIs. Second, we used self-reported behaviors to test criterion validity. Future research is encouraged to use objective measures of provider behaviors. Third, the five domains of this questionnaire were highly correlated in our data. Future research could consider refining the items with less similar wording across items to reduce method effect. Fourth, five TDF domains were not covered in the final questionnaire, including Behavioral regulation, Emotion, Goals, Memory, attention and decision processes, and Reinforcement. Future research should consider developing and validating new items measuring these domains. Finally, although consistent with declining and generally lower response rates of clinician surveys, our modest completion rate may introduce selection bias [51–53]. Future research should examine if the questionnaire shows similar psychometric properties in their target population.

## Conclusions

Using a national sample of clinicians, we conducted initial validation of a TDF-informed questionnaire assessing determinants of clinician adoption of novel CRC screening strategies using mt-sDNA as an example. Our evaluation showed that this questionnaire covers 5 domains with reasonable validity and good internal consistency. Future research should be undertaken to ascertain this instrument’s validity and reliability among GIs, its ability in predicting actual provider behaviors, and its generalizability to other CRC screening strategy contexts. Nonetheless, our findings suggest that data collected through this questionnaire can be useful for identifying barriers and facilitators to adoption of mt-sDNA in clinical practice and informing the selection of appropriate implementation strategies.

## Supplementary Information

**Additional file 1: Appendix 1.** Questionnaire items and theoretical domains

## Data Availability

The datasets used and/or analyzed during the current study are available from the corresponding author on reasonable request.

## Abbreviations

mt-sDNA: Multi-target stool DNA
CRC: Colorectal cancer
TDF: Theoretical domains framework
PCC: Primary care clinicians
GI: Gastroenterologist
CFA: Confirmatory factor analysis
HTMT: Heterotrait-Monotrait Ratio
WLSMV: Weighted least squares estimation
SRMR: Standardized root mean square residual
RMSEA: Root mean square error of approximation
CFI: Comparative fit index
MANOVA: Multivariate analysis of variance
ANOVA: Analysis of variance
