# Supplementary material for: Initial validation of a self-report questionnaire based on the Theoretical Domains Framework: determinants of clinician adoption of a novel colorectal cancer screening strategy
Source: Implement Sci Commun. 2021 Oct 19;2:119. doi: 10.1186/s43058-021-00221-x (PMC8527805; doi:10.1186/s43058-021-00221-x)
Supplement: Supplementary file 1 — Additional file 1: Appendix 1. Questionnaire items and theoretical domains [file 43058_2021_221_MOESM1_ESM.zip › Additional file 1.docx]

Appendix 1. Questionnaire items and theoretical domains

| **Initial TDF domain** | **Item ID** | **Item** | **Reason for removal** | **10-factor CFA** | **Cluster analysis** | **5-factor CFA** |
| --- | --- | --- | --- | --- | --- | --- |
| D1. Knowledge | D1-1 | I am aware that Cologuard is a CRC screening option for my average risk patients |  | D1. Knowledge | Cluster 5 | F1. Knowledge |
| D1. Knowledge | D1-2 | I understand that patients who receive a positive Cologuard result need to complete a follow-up diagnostic colonoscopy |  | D1. Knowledge | Cluster 5 | F1. Knowledge |
| D1. Knowledge | D1-3 | I understand that patients who receive a positive Cologuard result do not necessarily have CRC |  | D1. Knowledge | Cluster 5 | F1. Knowledge |
| D1. Knowledge | D1-4 | I understand which patients are appropriate for Cologuard |  | D1. Knowledge | Cluster 5 | F1. Knowledge |
| D1. Knowledge | D1-5 | I am aware that Cologuard should be repeated every 3 years for average risk patients |  | D1. Knowledge | Cluster 5 | F1. Knowledge |
| D1. Knowledge | D1-6 | I am aware of how Cologuard is used in my clinical practice |  | D1. Knowledge | Cluster 5 | F1. Knowledge |
| D1. Knowledge | D1-7 | I understand the differences between Cologuard and other stool-based CRC screening tests |  | D1. Knowledge | Cluster 5 | F1. Knowledge |
| D1. Knowledge | D1-8 | I understand how to assess whether my patients are at average risk for CRC |  | D1. Knowledge | Cluster 5 | F1. Knowledge |
| D4. Beliefs about capabilities | D4-1 | I have the necessary skills to order Cologuard for my patients |  | D4. Beliefs about capabilities | Cluster 5 | F1. Knowledge |
| D4. Beliefs about capabilities | D4-2 | I am confident that I understand when it is appropriate to offer Cologuard to my patients |  | D4. Beliefs about capabilities | Cluster 5 | F1. Knowledge |
| D2. Skills | D2-1 | I have been trained on how to order Cologuard for my patients |  | D2. Skills | Cluster 1 | F2. Skills |
| D2. Skills | D2-2 | It is easy to track and monitor patient adherence with Cologuard |  | D2. Skills | Cluster 1 | F2. Skills |
| D2. Skills | D2-3 | Ordering Cologuard for my patients is easy |  | D2. Skills | Cluster 1 | F2. Skills |
| D2. Skills | D2-4 | Ordering Cologuard for my patients doesn’t take a lot of time |  | D2. Skills | Cluster 1 | F2. Skills |
| D2. Skills | D2-5 | It is easy to receive Cologuard results |  | D2. Skills | Cluster 1 | F2. Skills |
| D3. Social/professional role and identity | D3-1 | I believe it is my responsibility to describe all available CRC screening options to patients, including Cologuard |  | D3. Social/professional role and identity | Cluster 2 | F3. Professional role and social influence |
| D3. Social/professional role and identity | D3-2 | I believe it is my responsibility to give patients a choice of using Cologuard for CRC screening along with other screening options |  | D3. Social/professional role and identity | Cluster 2 | F3. Professional role and social influence |
| D12. Social influences | D12-1 | Providers in my division/department believe it is their responsibility to describe all available CRC screening options to patients, including Cologuard |  | D12. Social influences | Cluster 2 | F3. Professional role and social influence |
| D12. Social influences | D12-2 | Providers in my division/department believe it is their responsibility to give patients a choice of using Cologuard for CRC screening along with other screening options |  | D12. Social influences | Cluster 2 | F3. Professional role and social influence |
| D12. Social influences | D12-3 | Providers in my division/department routinely describe all available CRC screening options to patients, including Cologuard |  | D12. Social influences | Cluster 2 | F3. Professional role and social influence |
| D12. Social influences | D12-4 | Providers in my division/department routinely give patients a choice of using Cologuard for CRC screening along with other screening options |  | D12. Social influences | Cluster 2 | F3. Professional role and social influence |
| D5. Optimism | D5-2 | I am optimistic that Cologuard is an effective CRC screening test |  | D5. Optimism | Cluster 3 | F4. Optimism, beliefs about consequences, and intentions |
| D5. Optimism | D5-3 | I am optimistic that patients will be more likely to complete Cologuard versus more invasive CRC screening tests |  | D5. Optimism | Cluster 3 | F4. Optimism, beliefs about consequences, and intentions |
| D5. Optimism | D5-1 | The evidence that supports the use of Cologuard for CRC screening is strong |  | D5. Optimism | Cluster 3 | F4. Optimism, beliefs about consequences, and intentions |
| D6. Beliefs about consequences | D6-1 | I believe that Cologuard will improve population level adoption of CRC screening |  | D6. Beliefs about consequences | Cluster 3 | F4. Optimism, beliefs about consequences, and intentions |
| D6. Beliefs about consequences | D6-2 | If I recommend Cologuard to patients, it will improve population health |  | D6. Beliefs about consequences | Cluster 3 | F4. Optimism, beliefs about consequences, and intentions |
| D6. Beliefs about consequences | D6-3 | I believe that Cologuard is an appropriate CRC screening test |  | D6. Beliefs about consequences | Cluster 3 | F4. Optimism, beliefs about consequences, and intentions |
| D8. Intentions | D8-1 | I would offer Cologuard to my patients for CRC screening |  | D8. Intentions | Cluster 3 | F4. Optimism, beliefs about consequences, and intentions |
| D8. Intentions | D8-2 | I intend to recommend Cologuard to my patients |  | D8. Intentions | Cluster 3 | F4. Optimism, beliefs about consequences, and intentions |
| D8. Intentions | D8-3 | I intend to promote the use of Cologuard among my colleagues |  | D8. Intentions | Cluster 3 | F4. Optimism, beliefs about consequences, and intentions |
| D8. Intentions | D8-4 | I support the use of Cologuard for average risk patients |  | D8. Intentions | Cluster 3 | F4. Optimism, beliefs about consequences, and intentions |
| D1. Knowledge | D1-11 | I am aware that Cologuard includes a patient navigation program to support test completion |  | D1. Knowledge | Cluster 4 | F5. Environmental context and resources |
| D1. Knowledge | D1-12 | I am knowledgeable about the features of the Cologuard patient navigation system |  | D1. Knowledge | Cluster 4 | F5. Environmental context and resources |
| D11. Environmental context and resources | D11-2 | The EMR system my practice uses makes it easy to receive Cologuard results |  | D11. Environmental context and resources | Cluster 4 | F5. Environmental context and resources |
| D11. Environmental context and resources | D11-3 | There are systems in place in my clinical practice to remind me that Cologuard is available as a CRC screening test |  | D11. Environmental context and resources | Cluster 4 | F5. Environmental context and resources |
| D11. Environmental context and resources | D11-4 | My patients often request Cologuard for CRC screening |  | D11. Environmental context and resources | Cluster 4 | F5. Environmental context and resources |
| D11. Environmental context and resources | D11-5 | The EMR system my practice uses makes it easy to order Cologuard |  | D11. Environmental context and resources | Cluster 4 | F5. Environmental context and resources |
| D11. Environmental context and resources | D11-6 | The care processes in place in my practice make it easy to follow patients who have been ordered a Cologuard test |  | D11. Environmental context and resources | Cluster 4 | F5. Environmental context and resources |
| D1. Knowledge | D1-9 | I am aware of Cologuard as a CRC screening test | Highly correlated (>.85) with D1-1 | D1. Knowledge |  |  |
| D1. Knowledge | D1-10 | I understand how to assess whether my patients are asymptomatic for CRC | Highly correlated (>.85) with D1-8 | D1. Knowledge |  |  |
| D5. Optimism | D5-4 | Cologuard represents a positive development in CRC screening | Highly correlated (>.85) with D6-1 | D5. Optimism |  |  |
| D6. Beliefs about consequences | D6-4 | I believe that use of Cologuard in my clinical practice will improve patient adherence to CRC screening recommendations | Highly correlated (>.85) with D6-2 and D6-5 | D6. Beliefs about consequences |  |  |
| D6. Beliefs about consequences | D6-5 | I believe that the availability of Cologuard as an option for CRC screening will improve the delivery of guideline concordant care in my clinical practice | Highly correlated (>.85) with D6-2 and D6-4 | D6. Beliefs about consequences |  |  |
| D10. Memory, attention and decision processes | D10-1 | I routinely describe all available CRC screening options to patients, including Cologuard | Measures behavior outcome instead of antecedent; Highly correlated (>.85) with D12-3 | D10. Memory, attention, and decision processes |  |  |
| D10. Memory, attention and decision processes | D10-2 | I routinely give patients a choice of using Cologuard for CRC screening along with other screening options | Measures behavior outcome instead of antecedent; Highly correlated (>.85) with D12-4 | D10. Memory, attention, and decision processes |  |  |
| D10. Memory, attention and decision processes | D10-3 | I routinely provide Cologuard for average risk patients before offering other screening options | Measures behavior outcome instead of antecedent; Highly correlated (>.85) with D12-9 | D10. Memory, attention, and decision processes |  |  |
| D10. Memory, attention and decision processes | D10-4 | I routinely only provide Cologuard for average risk patients once they have refused other screening options | Measures behavior outcome instead of antecedent | D10. Memory, attention, and decision processes |  |  |
| D11. Environmental context and resources | D11-1 | There are systems in place in my clinical practice to remind me to order CRC screening for my patients | Did not map onto the intended theoretical domain | D11. Environmental context and resources | Cluster 1 |  |
| D11. Environmental context and resources | D11-7 | There are systems in place in my clinical practice to remind me to order Cologuard screening for my patients | Highly correlated (>.85) with D11-3 | D11. Environmental context and resources |  |  |
| D12. Social influences | D12-5 | Providers in my division/department support the use of Cologuard for average risk patients | Did not map onto the intended theoretical domain | D12. Social influences | Cluster 3 |  |
| D12. Social influences | D12-6 | Most people whose opinion I value would support the use of Cologuard for CRC screening | Did not map onto the intended theoretical domain | D12. Social influences | Cluster 3 |  |
| D12. Social influences | D12-7 | Providers in my division/department believe that Cologuard is an appropriate CRC screening test | Did not map onto the intended theoretical domain | D12. Social influences | Cluster 3 |  |
| D12. Social influences | D12-8 | My colleagues are supportive of using Cologuard for CRC screening | Did not map onto the intended theoretical domain | D12. Social influences | Cluster 3 |  |
| D12. Social influences | D12-9 | Providers in my division/department routinely provide Cologuard for average risk patients before offering other screening options | Did not map onto the intended theoretical domain | D12. Social influences | Cluster 4 |  |
| D12. Social influences | D12-10 | Providers in my division/department routinely only provide Cologuard for average risk patients once they have refused other screening options | Did not map onto the intended theoretical domain | D12. Social influences | Cluster 4 |  |
| D7. Reinforcement |  |  |  |  |  |  |
| D9. Goals |  |  |  |  |  |  |
| D13. Emotion |  |  |  |  |  |  |
| D14. Behavioral regulation |  |  |  |  |  |  |
